# Cystic fibrosis: insights from zebrafish models

**DOI:** 10.1242/dmm.052856

**Published:** 2026-08-05

**Authors:** Sarahdja Cornélie, Laure Yatime, Georges Lutfalla, Stephen A. Renshaw, Audrey Bernut

**Affiliations:** ^1^ Laboratory of Pathogens and Host Immunity, CNRS, Inserm, Université de Montpellier, 34095 Montpellier, France; ^2^ The Bateson Centre, School of Medicine and Population Health, University of Sheffield, Sheffield S10 2TN, UK

**Keywords:** Cystic fibrosis, Zebrafish, CFTR, Innate immunity, Inflammation, Infections, Drug discovery

## Abstract

Cystic fibrosis (CF) is a severe, life-limiting genetic disorder caused by mutations in the *CFTR* gene, which lead to defective epithelial ion transport, abnormally thick mucus and multi organ dysfunction, predominantly affecting the lungs, pancreas and digestive system. Despite significant advances in patient care, the complex interplay between CFTR dysfunction, chronic infection and persistent inflammation remains a major therapeutic challenge. In this context, animal models are indispensable for elucidating the cellular and molecular mechanisms underlying CF pathogenesis and accelerating drug discovery. Here, we review the relevance of the zebrafish (*Danio rerio*) as a powerful and complementary preclinical model for CF research. In particular, we highlight the unique advantages of zebrafish, including its highly conserved innate immune system and optical transparency, which together enable *in vivo* visualization of host immune responses under CF-like conditions at subcellular resolution. We further summarize how Cftr-deficient zebrafish models have provided key insights into the increased susceptibility to CF-relevant pathogens, disease mechanisms affecting the pancreas and the reproductive system, and the deleterious neutrophil-driven inflammation that characterizes CF. Finally, we discuss the potential of the zebrafish model for the identification and validation of novel therapeutic strategies to treat infectious and inflammatory lung pathology in CF, and outline future directions to expand its translational impact in CF research.

## Teaser

**Summary:** This Review highlights how zebrafish models complement existing cystic fibrosis systems by enabling *in vivo* analysis of CFTR-dependent infection, inflammation, tissue damage and therapeutic responses.

## Introduction to cystic fibrosis

Cystic fibrosis (CF), the most common life-limiting autosomal recessive disorder among populations of Northern European ancestry, primarily affecting the lungs. It is caused by mutations in the gene encoding the CF transmembrane conductance regulator (CFTR) (Riordan et al., 1989). Among the >2000 CFTR variants identified, F508del is the most common disease-causing variant worldwide; however, many other variants contribute to disease and are commonly classified according to their functional consequences on CFTR synthesis, processing, trafficking, gating, conductance or protein stability (Mall et al., 2024). The protein is an atypical member of the ATP-binding cassette (ABC) transporter family of transmembrane proteins and functions as a cAMP-regulated anion channel. By mediating the transport of chloride (Cl^−^) and bicarbonate (HCO₃^−^) ions across epithelial surfaces, CFTR regulates pH values, as well as epithelial fluid secretion and mucosal hydration, processes that are central to CF pathogenesis (Mall et al., 2024). CFTR is expressed predominantly at the apical membrane of epithelial cells lining the airways, pancreatic ducts, intestine, hepatobiliary tract, sweat glands and male reproductive tract (Mall et al., 2024). CFTR is also present in several immune cell types, including macrophages, neutrophils and lymphocytes, supporting roles beyond epithelial ion transport (Mall et al., 2024). This pattern of expression is consistent with the multisystem involvement of CF, although progressive lung disease remains the primary cause of morbidity and mortality in individuals with CF (Elborn, 2016).

In the airway epithelium, defective CFTR-mediated ion transport disrupts airway surface liquid homeostasis, leading to increased Na^+^ and H_2_0 reabsorption (Boucher, 2007; Mall et al., 2024). This results in the accumulation of dehydrated mucus and impaired mucociliary clearance. As a consequence, microorganisms are inefficiently cleared from the airways, predisposing patients to recurrent and eventually chronic infections (Mall et al., 2024; Morrison et al., 2019). CF-related infections involve a characteristic spectrum of pathogens, including *Staphylococcus aureus*, *Haemophilus influenzae*, *Pseudomonas aeruginosa*, members of the *Burkholderia cepacia* complex and non-tuberculous mycobacteria (NTM) (Hatziagorou et al., 2020). In response to persistent infections, epithelial and immune cells release pro-inflammatory mediators, such as reactive oxygen species (ROS) and cytokines, driving excessive neutrophil recruitment and activation. This self-amplifying inflammatory response contributes to progressive airway tissue damage and loss of lung function (Cantin et al., 2015; Elborn, 2016).

Susceptibility to infection in CF has long been attributed primarily to mucus obstruction and defective mucociliary clearance, and chronic inflammation has been viewed as a secondary consequence of ineffective pathogen clearance. However, mucociliary dysfunction alone does not fully account for the CF lung phenotype. Notably, other disorders with similarly severe mucus stasis, such as primary ciliary dyskinesia, exhibit markedly different clinical manifestations (Cohen-Cymberknoh et al., 2014). Accumulating evidence now indicates that CFTR dysfunction also directly impairs host innate immune responses, leading to altered bacterial clearance, excessive inflammatory responses and defective tissue repair, all of which contribute to CF-associated lung disease (Lara-Reyna et al., 2020). However, the precise mechanisms by which CFTR regulates immune and inflammatory pathways remain incompletely understood.

Over the past decades, CF prognosis has improved considerably, with median life expectancy now exceeding 40−50 years in many countries, largely due to advances in multidisciplinary care and disease-modifying therapies (Bell et al., 2020). Current therapeutic strategies include airway clearance techniques, antibiotic therapy for exacerbations and chronic infection, anti-inflammatory treatments, pancreatic enzyme replacement therapy and nutritional support (Burgel et al., 2024; Mall et al., 2024). More recently, CFTR modulator therapies, such as potentiators and correctors, including deutivacaftor, elexacaftor, ivacaftor, lumacaftor, tezacaftor and vanzacaftor, have transformed disease management by targeting the underlying protein defect, significantly improving lung function and quality of life for many patients (Middleton et al., 2019). However, these therapies are not effective for all CFTR variants, are not universally accessible and do not fully reverse established lung damage.

Although CF patient care has improved substantially, chronic infections, persistent inflammation and lung damage remain critical unmet clinical needs, particularly in patients with advanced disease or those not eligible for CFTR modulators (Graeber and Mall, 2023; Harvey et al., 2022; Keown et al., 2020). The development and use of suitable experimental models are, therefore, essential to unravel the complex mechanisms driving CF pathogenesis and to facilitate the discovery of more effective therapeutic strategies.

## Overview of cystic fibrosis models

A wide range of human cellular systems and animal models has been developed and used to decipher the pathophysiology of CF, and to evaluate new therapeutic approaches. Each model has distinct advantages and limitations, and the choice of model depends on the biological question being addressed – ranging from CFTR channel function to infection, inflammation, tissue remodelling or therapeutic screening (for more details, see recent reviews by Grubb and Livraghi-Butrico, 2022; McCarron et al., 2021; Rosen et al., 2018; Semaniakou et al., 2019).

Human cell lines and primary cells derived from CF patients have been invaluable for dissecting CFTR-dependent cellular mechanisms and for testing CFTR-modulating therapies (Gruenert et al., 2004). However, these *in vitro* systems are inherently limited in their ability to distinguish the direct effects of CFTR dysfunction from secondary disease processes, including chronic infection, inflammation and tissue remodelling. In addition to conventional cell lines, advanced three-dimensional culture systems, such as patient-derived organoids have emerged as powerful tools for investigating CFTR function in a patient-specific context (de Poel et al., 2020; Sachs et al., 2019). These intestinal, airway and pancreatic organoids recapitulate key features of epithelial architecture and CFTR-dependent ion transport and have proven particularly valuable for predicting individual responses to CFTR modulators, notably because they preserve the CFTR genotype of the patient (Berkers et al., 2019; de Poel et al., 2020; Sachs et al., 2019). Despite their increased physiological relevance, organoids remain restricted mainly to epithelial compartments and do not capture the full complexity of CF-associated infections and inflammatory responses, which rely on dynamic interactions between immune cells, vascular components and the extracellular matrix.

To address these limitations, several mammalian models of CF have been generated, most notably in mice. Mouse models have been instrumental in advancing our understanding of CFTR biology and epithelial ion transport, and in the evaluation of CFTR-targeted therapies (McCarron et al., 2021). Nevertheless, important physiological differences between rodents and humans constrain their translational relevance. Mice carrying disrupted *Cftr* alleles typically develop intestinal obstruction resembling that observed in individuals with CF but, generally, do not exhibit spontaneous lung disease or significant pancreatic pathology comparable to human (Davidson and Rolfe, 2001; McCarron et al., 2021; Semaniakou et al., 2019). Species-specific differences in airway architecture, airway surface liquid composition and microbial colonization are thought to underlie the absence of a characteristic CF-like pulmonary phenotype in mice (McCarron et al., 2021; Rosen et al., 2018; Semaniakou et al., 2019). To partially overcome these limitations, mouse models overexpressing sodium channel epithelial 1 subunit beta (SCNN1B; also known as βENaC) were developed, which reproduce several hallmarks of CF lung disease, including airway dehydration, mucus obstruction and neutrophilic inflammation, despite the absence of CFTR mutations (Mall et al., 2004). These models have provided important mechanistic insights into CF-like airway pathology but remain complementary to CFTR-deficient models.

To better recapitulate human disease, CF models of larger mammals have subsequently been developed, i.e. of pigs (Rogers et al., 2008), ferrets (Sun et al., 2010), rats (Tuggle et al., 2014), sheep (Fan et al., 2018) and rabbits (Le Bras, 2021). Among these models, CF pigs and ferrets most closely reproduce key features of human CF, including spontaneous airway infection, inflammation and pancreatic pathology. However, these systems are associated with substantial limitations, including high costs of animal husbandry, technical challenges in genetic manipulation, fragility of the animals, limited access to the earliest stages of disease and significant ethical constraints. Furthermore, they do not readily allow routine intravital imaging of host−pathogen interactions and inflammatory responses at single-cell resolution within intact tissues.

Importantly, CF does not occur naturally in any animal species. Although the *CFTR* gene is highly conserved across vertebrates, no spontaneous mutations leading to CF-like disease have been identified so far, and all existing models rely on targeted genetic disruption of *CFTR* orthologues (Grubb and Livraghi-Butrico, 2022; McCarron et al., 2021; Semaniakou et al., 2019). This evolutionary conservation, together with the relatively high carrier frequency of CFTR mutations in human populations – estimated to be approximately one in 25 among people of Northern European ancestry (Farrell, 2008) – has led to the hypothesis that CFTR heterozygosity has been maintained by selective advantage (Poolman and Galvani, 2007). In particular, partial reduction of CFTR function has been proposed to reduce susceptibility to selected infectious pressures, including severe secretory diarrhoeal diseases, such as cholera or typhoid fever and, possibly, tuberculosis (Bosch et al., 2017; Poolman and Galvani, 2007). However, these hypotheses remain debated, and no single selective pressure fully explains the present-day distribution of CFTR variants.

Collectively, although *in vitro* and mammalian models have been indispensable for modelling specific aspects of CF pathogenesis, logistical, technical and ethical constraints limit their utility for mechanistic studies of infection and inflammation, and for therapeutic discovery. In this context, small non-mammalian models provide complementary advantages. Invertebrate models, such as *Drosophila melanogaster*, have provided insights into conserved pathways related to epithelial ion transport, host−pathogen interactions and innate immunity (Touré et al., 2023). Notably, a functional *Drosophila* Cftr orthologue has been identified, enabling genetic studies of CFTR-related epithelial physiology, although this model cannot reproduce the organ-level manifestations of human CF (Kim et al., 2020; Touré et al., 2023). In parallel, vertebrate models such as *Danio rerio* (zebrafish) combine experimental accessibility, genetic tractability and the ability to investigate CFTR-dependent processes *in vivo* within a living organism. The following sections will focus on the zebrafish model, highlighting its unique advantages for studying CF pathophysiology, particularly in the context of infection, inflammation and for accelerating therapeutic discovery.

### A fish called *Danio rerio*: the relevance of zebrafish in cystic fibrosis research

The zebrafish, a small freshwater teleost fish, has emerged as a powerful non-mammalian model organism for studying human diseases, with growing impact in translational research (Lieschke and Currie, 2007). Many disease-associated pathways, including those governing aetiology and progression, are evolutionarily conserved between zebrafish and humans, making this model particularly relevant for investigating complex pathophysiological processes. Compared with most mammalian models, zebrafish offers major practical advantages, including external fertilisation, rapid embryonic development, early sexual maturity, high reproductive capability, low maintenance cost and reduced space requirements. Importantly, in many jurisdictions, zebrafish embryos and larvae at ≤5 days post fertilisation are not subject to animal experimentation regulations, providing a supportive platform for preclinical research in accordance with the 3Rs principles (Strähle et al., 2012).

**Fig. 1.**
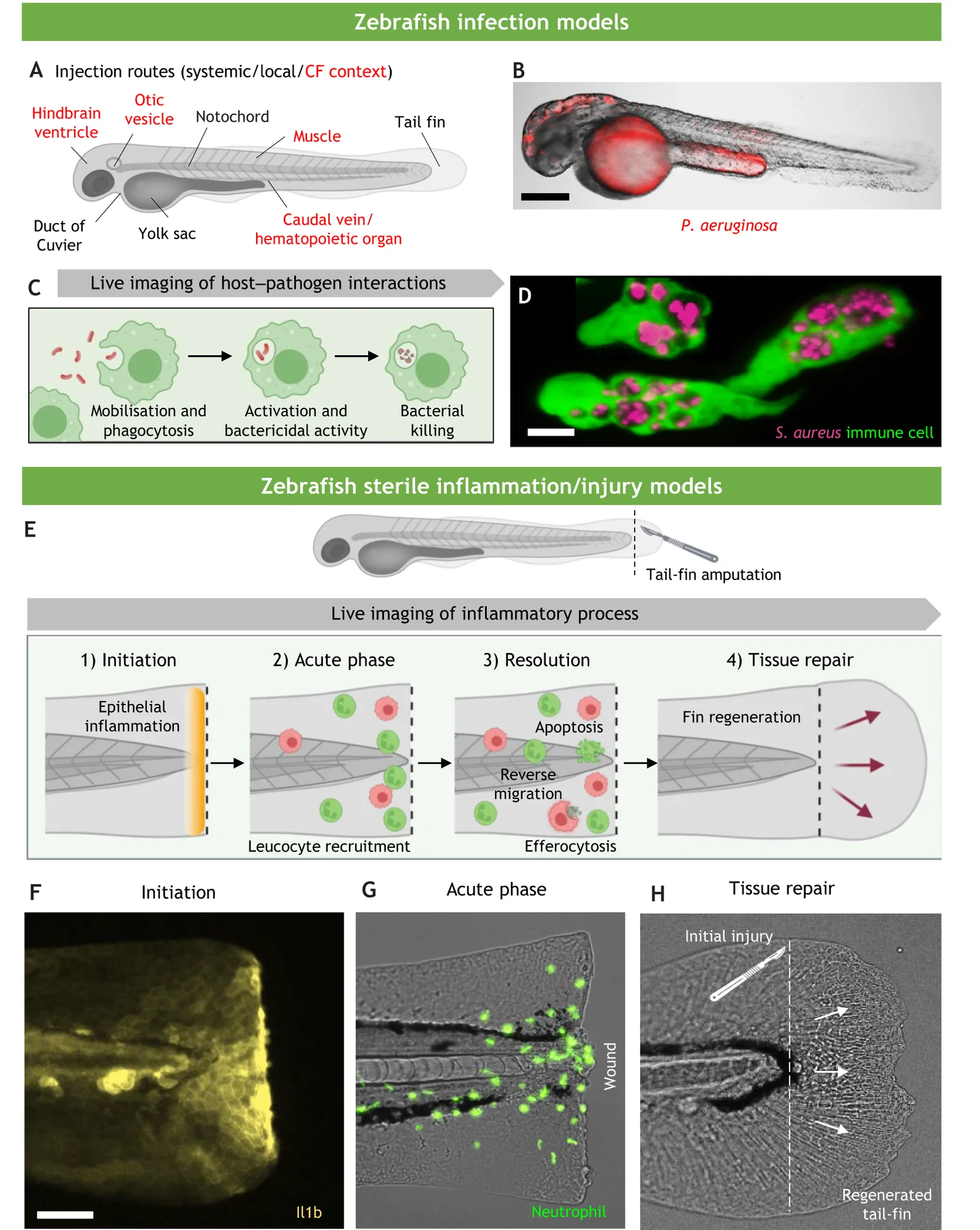
**Zebrafish larval models for *in vivo* visualization of infectious and aseptic inflammatory processes.** (A-D) Modelling infectious processes in zebrafish larvae. (A) Schematic overview of the most commonly used microinjection routes in zebrafish larval infection models in normal or CF contexts. In zebrafish infection models, pathogens are typically introduced into accessible compartments such as the bloodstream, usually via the caudal vein or duct of Cuvier, the hindbrain ventricle, the otic vesicle or muscle tissue (Torraca and Mostowy, 2018). Importantly, the site of injection determines the nature of the infection: vascular injections generally promote systemic dissemination, whereas localised injections enable spatially confined analyses of host−pathogen interactions, in particular epithelial activation and immune cell recruitment, while still supporting progressive infection. The tropism and extent of dissemination are also dose- and pathogen-dependent (Gomes and Mostowy, 2020). As such, the injection site does not inherently bias outcomes but instead defines the experimental context and should be chosen according to the specific biological question. (B) Real-time *in vivo* imaging of an infectious process in zebrafish larvae infected with fluorescent *P. aeruginosa* (scale bar: 500 m). (C) The use of transgenic zebrafish lines with fluorescently labelled innate immune cells (e.g. macrophages and neutrophils), combined with fluorescent pathogens, enables high-resolution *in vivo* analysis of host−pathogen interactions during an infectious process. (D) Confocal micrograph showing a professional phagocyte (neutrophil, green) that has phagocytosed *S. aureus* (red) (scale bar: 5 m). (E-H) Modelling aseptic inflammation in zebrafish larvae using a caudal fin injury model. In zebrafish larvae, non-infectious inflammatory processes can be investigated using caudal fin amputation as a model of aseptic tissue injury. (E) Schematic representation of the inflammatory cascade triggered by epithelial injury following tail-fin amputation. The conserved kinetics of human inflammatory responses is recapitulated in four phases. (1) Initiation phase, mainly characterized by epithelial release of early pro-inflammatory mediators (e.g. Ca^2+^, ROS and cytokines), promoting leukocyte recruitment to the wound. (2) Acute inflammatory phase, marked by robust infiltration of innate immune cells, including neutrophils and macrophages, within the injured tissue. (3) Resolution phase, involving cessation of leukocyte recruitment and active clearance of inflammatory cells through processes such as reverse migration, apoptosis and efferocytosis, leading to restoration of tissue homeostasis, and (4) initiation of tissue repair, which in zebrafish culminates in complete caudal fin regeneration. (F) Representative visualization of Il1b production by epithelial cells during the acute inflammatory phase following tail fin amputation (scale bar: 100 m). (G) Dynamics of neutrophil recruitment to the wound site. (H) Regeneration of the caudal fin (arrows) during the regenerative phase following fin amputation. Schematic illustrations were created in BioRender by Bernut, A (2026); https://BioRender.com/mjktjwo. This figure was sublicensed under CC-BY 4.0 terms.

Genomic analyses have revealed a high degree of conservation between zebrafish and humans: ∼70% of human genes have at least one zebrafish orthologue, and >80% of genes associated with human diseases are conserved in the zebrafish genome (Howe et al., 2013). The extensive accessibility of zebrafish genetic resources and databases (e.g. ZFIN), combined with efficient genome-editing tools, such as CRISPR/Cas9 and morpholino-based approaches (Daniel et al., 2023; Li et al., 2021; Stainier et al., 2017), further enhances the versatility of this model. As a vertebrate organism, zebrafish possess an immune system that is closely homologous to that of humans, particularly with respect to innate immunity (Renshaw and Trede, 2012). In addition, the optical transparency of embryos and larvae allows non-invasive, real-time monitoring of infectious and inflammatory processes following aseptic or pathogen-induced tissue injury. When combined with high-resolution microscopy, fluorescent reporter immune cell lines and labelled pathogens, zebrafish allow host−pathogen interactions and innate immune responses to be visualised at cellular and subcellular resolution across tissues within the complexity of a whole animal, and throughout the course of infection (Fig. 1A-D) or inflammation (Fig. 1E-H) (Gomes and Mostowy, 2020; Leiba et al., 2023; Torraca et al., 2014).

Zebrafish are now well established as a valuable model for studying the virulence of human pathogens, including those relevant to pulmonary disease. Although zebrafish do not possess lungs, infection models in this organism recapitulate key features of host−pathogen interactions and innate immune responses relevant to respiratory infections (Torraca and Mostowy, 2018). Notably, zebrafish infection models have yielded important insights into the pathogenic mechanisms of several CF-associated bacteria, especially *P. aeruginosa* (Clatworthy et al., 2009), *S. aureus* (Prajsnar et al., 2008), *B. cenocepacia* (Vergunst et al., 2010) and the NTM *Mycobacterium abscessus* (Bernut et al., 2014, 2016). Furthermore, zebrafish larvae respond to pharmacological treatments at physiologically relevant dose ranges, making them a valuable vertebrate platform for evaluating drug efficacy and toxicity *in vivo* (Cassar et al., 2020). These approaches have been widely applied to discover anti-infective and anti-inflammatory drugs, taking advantage of rapid compound exposure, whole-organism readouts and compatibility with medium- to high-throughput screening (Habjan et al., 2024; Zanandrea et al., 2020).

**Fig. 2.**
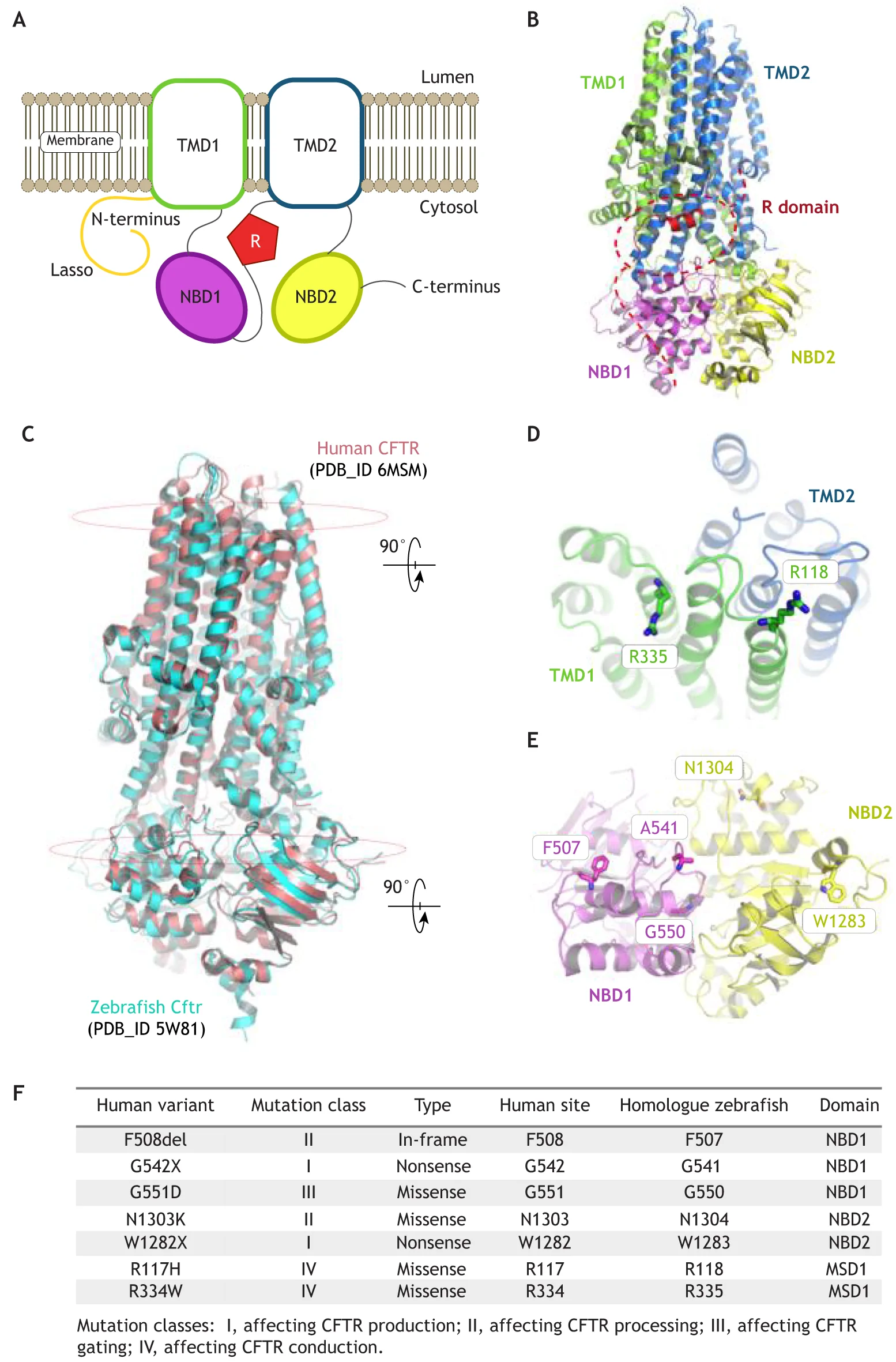
**Zebrafish and human CFTR protein structures are highly homologous, with conserved amino acid residues at positions most frequently mutated in CF.** (A) Schematic representation of the domain organization of the CFTR ion channel, showing the N-terminal lasso motif (yellow) anchored into the cell membrane, transmembrane domains 1 (TMD1, green) and 2 (TMD2 (blue), cytosolic nucleotide-binding domains 1 (NBD1, purple) and 2 (NBD2 (yellow), and the regulatory domain (R, red). (B) Quaternary structure of human CFTR obtained by using cryo-electron microscopy (cryo-EM) images at 3.2 Å resolution (PDB_ID 6MSM; Zhang et al., 2018). Color coding as in A. Notice that in all available CFTR structures, the position of the R domain is – due to its high flexibility – largely unresolved; its approximate position here is encircled by a dashed line (red), the dashed line protruding from the circle indicates the connection between the R domain and the NB1D and TMD2 domains. (C) Overlay of human and zebrafish quaternary CFTR protein structures obtained by using cryo-EM images. Human CFTR is shown in red (PDB_ID 6MSM; Zhang et al., 2018), zebrafish Cftr in blue; PDB_ID 5W81; Zhang et al., 2017). The overall root-mean-square-deviation on core Cα atoms between the two structures is 1.9 Å, which highlights the high degree of structural conservation between the two channels. The two regions where most mutated residues are found are indicated with red circles. (D,E) Parts of the quaternary protein structure of zebrafish Cftr showing the position of amino acid residues that correspond to those most frequently mutated in human CFTR, on the luminal side of the ion channel (D) or on the upper part of the NBD1 and NBD2 domains, facing the transmembrane domain (E). All residues are conserved in zebrafish Cftr, except human G551, which is replaced by A550 (conservative mutation) (see panel F). (F) List of clinically relevant CFTR variants and sites in human and their respective zebrafish homologues, as well as their respective mutation classes I–IV and domain locations. These examples were selected for their clinical relevance, relative frequency and suitability for mapping onto conserved CFTR/Cftr residues as shown in D and E.

The relevance of zebrafish to CF research is further underscored by the high degree of conservation of the Cftr protein. The first atomic structure of CFTR in human was solved by using zebrafish protein (Zhang and Chen, 2016). Zebrafish Cftr exhibits moderate sequence similarity with human CFTR (∼56% amino acid identity), while retaining a highly conserved domain organisation (Liu et al., 2017) (Fig. 2A-C). Importantly, 42 of the 46 CF-causing missense mutation sites analysed are identical between human CFTR and zebrafish Cftr. Moreover, deletion of amino acid phenylalanine (F) at position 508 (F508) that yields F508del, the most common CF-causing variant in humans is conserved as F507 in zebrafish Cftr, thereby supporting the relevance of zebrafish for mapping clinically relevant CFTR residues (Fig. 2D-F). Functional studies have shown that zebrafish Cftr and human CFTR protein share key properties essential for channel gating and regulation (Liu et al., 2017; Zhang and Chen, 2016). In line with this conservation, the CFTR potentiator ivacaftor also enhances zebrafish Cftr channel activity (Laselva et al., 2019). By contrast, *in vitro* assessment of the corrector lumacaftor failed to rescue misprocessing of mutant Cftr as it does in patients with CF (Laselva et al., 2019). As in mammals, Cftr plays a critical role in regulating fluid secretion and mucosal homeostasis in zebrafish exocrine organs. Loss of Cftr function disrupts inflation of Kupffer's vesicle, a transient ciliated structure essential for left-right patterning during early development, highlighting the importance of Cftr-mediated fluid transport in this model (Bernut et al., 2020; Navis et al., 2013). The tissue-specific expression pattern of *cftr* is also conserved between zebrafish and humans, with expression detected in the kidney, liver, spleen, pancreas and intestine (Navis and Bagnat, 2015; Phennicie et al., 2010). Consistent with observations in human systems, *cftr* expression in zebrafish, is not restricted to epithelial surfaces but is also detected in innate immune cells, including macrophages and neutrophils (Bernut et al., 2019; Phennicie et al., 2010), supporting a direct role for CFTR in immune function.

### Overview of current zebrafish models of CF

The first genetically modified zebrafish model of CF was generated by Bagnat's group using TALEN-mediated gene targeting of exon 6 of *cftr*, producing stable loss-of-function alleles (Navis et al., 2013). Subsequently, other homozygous Cftr-null zebrafish models were developed by using CRISPR-Cas9 technology (Bernut et al., 2020; Liao et al., 2018; Sun et al., 2018), expanding the genetic toolkit available to interrogate Cftr-dependent processes *in vivo*. These stable genetic models provide robust and reproducible systems to study CFTR loss-of-function across developmental stages and tissues. However, as zebrafish models carrying patient-specific CFTR mutations have not yet been developed, they primarily model complete CFTR deficiency and do not capture the diversity of mutation-specific effects observed in patients.

In parallel, antisense morpholino knockdown remains widely used in zebrafish for transient reduction of gene expression. Injection of *cftr*-targeting morpholinos at the one-cell stage, including translation- and splice-blocking morpholinos, generates *cftr*-depleted larvae (*cftr* morphants) (Bernut et al., 2019; Phennicie et al., 2010), enabling analysis of early consequences of Cftr loss of function within approximately one week without the need to generate stable mutant lines. Importantly, several studies report that *cftr* morphants reproduce key phenotypes observed in stable *cftr* mutants, supporting their utility as a complementary approach (Bernut et al., 2019; Bernut et al., 2020; Le Moigne et al., 2022; Weimann et al., 2024). While morpholino-based strategies offer significant advantages in terms of speed and experimental flexibility, their transient nature and potential off-target effects should be taken into consideration when interpreting results, particularly in comparison with stable genetic models.

Finally, pharmacological inhibition provides an additional, reversible method to phenocopy CFTR dysfunction *in vivo*. In zebrafish, Cftr activity can be inhibited using CFTRinh-172 (Bagnat et al., 2010; Dasgupta et al., 2018; Liao et al., 2018; Liu et al., 2020; Roxo-Rosa et al., 2015), a channel blocker commonly employed in human *in vitro* and mammalian systems. Pharmacological approaches allow temporal control of CFTR inhibition and are particularly useful for acute studies; however, they may be limited by compound specificity, stability and potential off-target effects.

To clarify the strengths, limitations and contributions of the different approaches used in the field, Table 1 summarizes current zebrafish CF models. It highlights how these complementary approaches have advanced the study of Cftr function, including developmental and extra-pulmonary phenotypes, infection susceptibility, inflammatory dysregulation, tissue repair and therapeutic responses.

**Table 1.** Overview of zebrafish CF models: approaches, phenotypes and therapeutic testing

CF model	Model/contribution	Main reported CF-related phenotypes/mechanisms	Evaluation of therapy	Key references
*cftr* TALEN mutants	TALEN targeting of *cftr* exon 6. Heritable loss-of-function alleles.	Defective Kupffer's vesicle inflation.
		Defective left-right patterning; pancreatic ductal mucus accumulation. Exocrine pancreatic destruction. Pancreatic neutrophilic inflammation. Increased susceptibility to *M. abscessus*, i.e. reduced larval survival.	Mainly mechanistic validation. Limited therapeutic testing.	Bernut et al., 2019; Navis and Bagnat, 2015; Navis et al., 2013
*cftr* CRISPR-Cas9 mutants	CRISPR-Cas9 targeting of coding *cftr*. Heritable frameshift/deletion loss-of-function alleles.	Impaired primordial germ cell migration. Reproductive defects. Haematopoietic defects. Sterile hyperinflammation, i.e. excessive epithelial and neutrophil responses. Impaired tissue repair; cardiac developmental defects.	Mainly mechanistic validation. Limited therapeutic testing.	Bernut et al., 2020; Liao et al., 2018; Liu et al., 2020; Sun et al., 2018; Weimann et al., 2024
Splice-blocking *cftr* morpholino	Transient disruption of *cftr* splicing.	Increased susceptibility to *M. abscessus, M. fortuitum* and *P. aeruginosa*, i.e. increased bacterial burden and reduced larval survival. Defective Nox2-dependent antimicrobial ROS production. Reduced Nrf2 activity. Excessive VGCC-dependent epithelial Ca^2+^ and excessive DUOX-dependent epithelial ROS after injury. Excessive and persistent neutrophilic inflammation. Defective neutrophil apoptosis and reverse migration. Altered cytokine responses. Impaired tissue repair and remodelling.	Roscovitine worsened *M. abscessus* infection by impairing ROS-dependent neutrophil recruitment. Roscovitine-/tanshinone IIA-promoted neutrophil apoptosis/reverse migration and improved repair. Curcumin/NRF2 activation reduced epithelial ROS, cytokines and tissue damage. Verapamil reduced epithelial Ca^2+^/ROS signalling and neutrophil recruitment. Phage therapy and phage-antibiotic combinations reduced *P. aeruginosa* and mycobacterial burden.	Bernut et al., 2019, 2020; Cafora et al., 2019, 2021, 2025; Cornélie et al., 2025 preprint; Johansen and Kremer, 2020; Johansen et al., 2021; Le Moigne et al., 2022; Leon-Icaza et al., 2025; Phennicie et al., 2010; Weimann et al., 2024
Pharmacological Cftr inhibition	Reversible and temporal inhibition with CFTRinh-172.	Defective Kupffer's vesicle inflation. Reduction of Cftr channel activity. Disruption of Cftr-dependent epithelial/developmental functions. Cardiac developmental abnormalities.	Mainly used to phenocopy Cftr dysfunction and define temporal requirements of Cftr activity.	Bagnat et al., 2010; Dasgupta et al., 2018; Liao et al., 2018; Liu et al., 2020; Roxo-Rosa et al., 2015

Taken together, these approaches offer powerful and versatile *in vivo* systems to interrogate CFTR function, yet remain largely restricted to modelling CFTR loss-of-function, highlighting the need for next-generation models that better reflect the diversity of CFTR dysfunction observed in patients.

## Lessons from zebrafish models of cystic fibrosis: bridging basic science and translational applications

Zebrafish models with impaired Cftr function reproduce different phenotypes that mirror key aspects of human CF. Reported manifestations include pancreatic destruction and pancreatitis-like pathology (Navis and Bagnat, 2015), haematopoietic defects consistent with anaemia (Sun et al., 2018), infertility linked to impaired primordial germ cell migration (Liao et al., 2018), increased susceptibility to CF-relevant pathogens (Bernut et al., 2019; Johansen and Kremer, 2020; Phennicie et al., 2010; Weimann et al., 2024) and aberrant inflammation with defective tissue repair/remodelling (Bernut et al., 2020; Cornélie et al., 2025 preprint; Leon-Icaza et al., 2025). Together, these studies position zebrafish as a uniquely accessible vertebrate system to dissect CFTR-dependent mechanisms *in vivo*, particularly at the intersection of infection, innate immunity and inflammatory tissue damage.

### Zebrafish models of cystic fibrosis: recapitulating innate immune dysregulation and beyond

#### Use of zebrafish to model CF-related infections

People with CF display high susceptibility to airway infections (Mall et al., 2024; Morrison et al., 2019). While mucociliary dysfunction clearly contributes, accumulating evidence indicates that mutations in *CFTR* also lead to defective bactericidal activity in immune cells, but the underlying mechanisms remain incompletely defined. Zebrafish provide a powerful opportunity to study infectious processes and interrogate host−pathogen interactions *in vivo*. Accordingly, several Cftr-depleted zebrafish models have been used to investigate why CFTR dysfunction predisposes to infection.

To date, spontaneous infections have not been established in CF zebrafish; however, upon experimental challenge (Fig. 1A), Cftr-depleted larvae show increased susceptibility to CF-relevant pathogens. In these models, infections are typically induced systemically and often progress to rapid disseminated infection, reflecting an impaired ability of the innate immune system to control bacterial spread (Bernut et al., 2019; Johansen and Kremer, 2020; Phennicie et al., 2010; Weimann et al., 2024). The outcomes include higher bacterial burdens and premature mortality following infection with *P. aeruginosa* (Phennicie et al., 2010; Weimann et al., 2024) and several NTM, including *M. abscessus* complex and *Mycobacterium fortuitum* (Bernut et al., 2019; Johansen and Kremer, 2020).

**Fig. 3.**
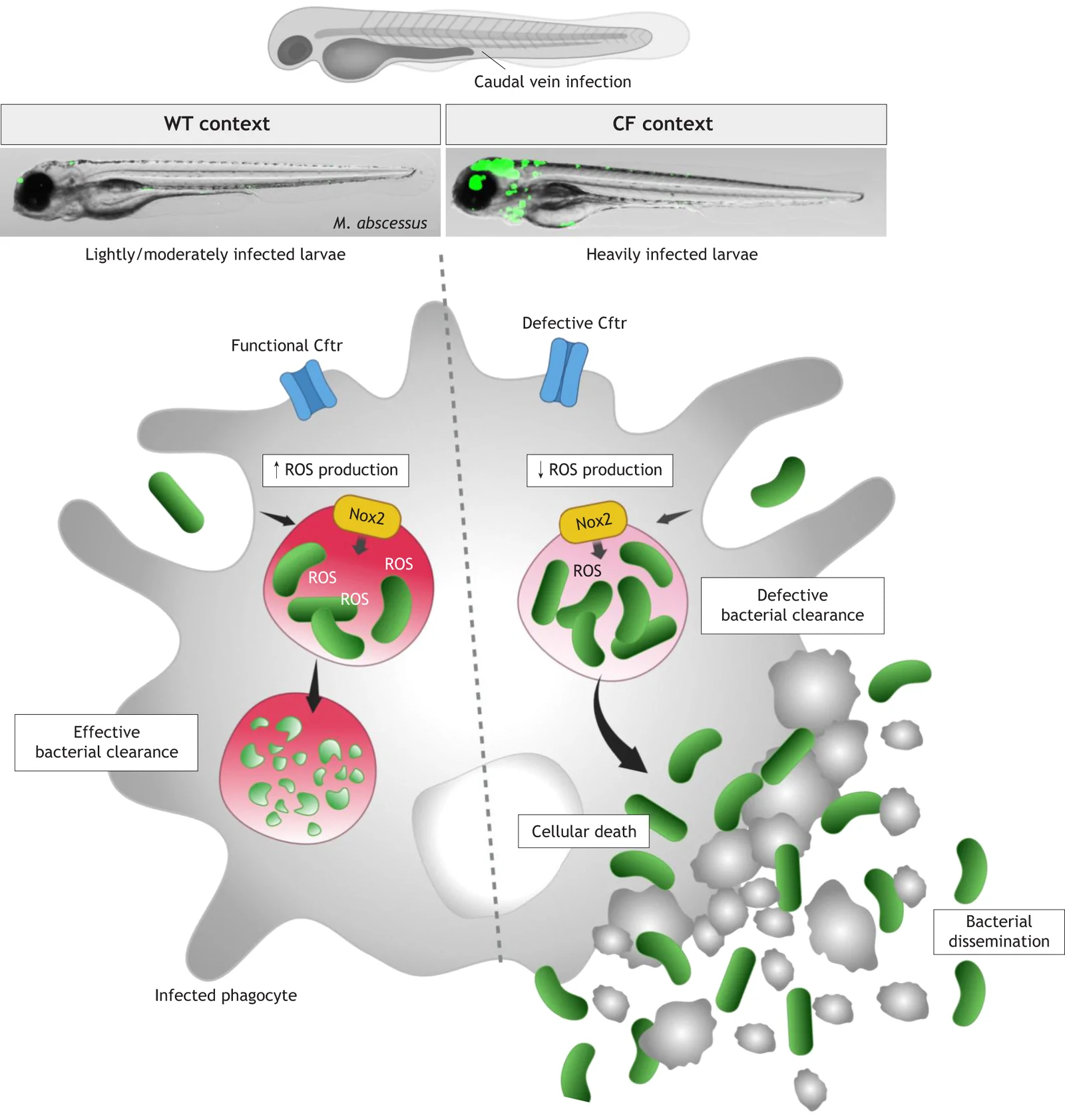
**Hypersusceptibility to infections and impaired bactericidal activity revealed by CF zebrafish models.** Schematic overview illustrating how Cftr dysfunction compromises host defence in zebrafish. Loss of Cftr disrupts Nox2-dependent ROS production in macrophages, resulting in defective intracellular bacterial killing. This impairment leads to failure of effective bacterial killing, uncontrolled bacterial proliferation and, ultimately, increased mortality in zebrafish larvae. Schematic illustrations were created in Biorender by Bernut, A (2026); https://BioRender.com/dt603w9. This figure was sublicensed under CC-BY 4.0 terms.

In the context of *M. abscessus* infections, Bernut et al. showed that Cftr deficiency compromises bacterial killing by professional phagocytes, in part through defective Nox2-dependent ROS production (Fig. 3), a critical host defence mechanism against intracellular bacteria (Bernut et al., 2019). These findings are consistent with previous reports showing altered oxidative responses in CF patient-derived macrophages (Assani et al., 2017). In these human CF macrophages, defective Ca^2+^-dependent PKC activation of NADPH oxidase impaired oxidative antimicrobial responses (Assani et al., 2017). In zebrafish, the failure in oxidative defence impairs the formation and stability of protective granulomas, facilitating extracellular expansion via mycobacterial cord formation, which drives acute infection (Bernut et al., 2014). These findings established a mechanistic link between CFTR dysfunction and impaired immunity against *M. abscessus*. More recently, Weimann et al. described how *P. aeruginosa* has evolved into a major human pathogen over the past 200 years (Weimann et al., 2024). In their work, the authors combined CF zebrafish infection models with analyses of human CF macrophages to propose that *P. aeruginosa* lineages with CF affinity exploit CF-associated immune defects as a route to niche specialization and epidemic success. Notably, bacteria carrying a mutation of the transcription factor DksA1, whose replication is altered under oxidative stress conditions regained the ability to proliferate in the CF zebrafish model (Weimann et al., 2024), suggesting defective oxidative defences in this model, consistent with findings from Bernut et al. and the broader literature on CFTR-dependent antimicrobial ROS responses (Assani et al., 2017; Bernut et al., 2019).

These observations also suggest that the contribution of Cftr to innate immunity is pathogen dependent. While Cftr-deficient zebrafish show enhanced susceptibility to several CF-associated bacteria, data indicate potentially divergent responses to *M. marinum*, a close genetic relative of the human pathogen *Mycobacterium tuberculosis* (Bernut et al., 2019; Cornelie et al., 2024). In this model, Cftr-deficient larvae appear less susceptible to *M. marinum*, showing increased survival rate and lower global and intramacrophage bacterial burdens. Such pathogen-specific effects echo evolutionary hypotheses proposing that CFTR variants may have been shaped by past infectious pressures, including tuberculosis-related selection (Bosch et al., 2017; Poolman and Galvani, 2007).

Collectively, zebrafish studies strengthen the concept that CFTR contributes to ROS-mediated antimicrobial immunity, helping to explain why CFTR dysfunction predisposes to particular pathogens beyond the effects of mucus stasis alone. Beyond oxidative killing, other antibacterial defence mechanisms have been reported to be altered in CF immune cells, including defects in phagosome maturation and phagolysosomal fusion (Di et al., 2006), and impaired autophagy (Flores-Vega et al., 2021). These processes remain incompletely explored in zebrafish CF models and represent promising avenues for future studies to further dissect CFTR-dependent immune dysfunction *in vivo*.

#### CF zebrafish exhibit an overactive and damaging inflammatory phenotype

Excessive and persistent neutrophil-predominant inflammation is a characteristic of CF-related lung disease (Cantin et al., 2015; Laval et al., 2016). Although this has often been viewed as a secondary consequence of chronic infection, inflammatory abnormalities can be detected early, sometimes preceding detectable infection, thus supporting the idea that CFTR dysfunction may directly disrupt inflammatory responses (Verhaeghe et al., 2007). The importance of this intrinsic characteristic in the onset of inflammation in CF airways is still debated. Zebrafish models of aseptic inflammation have been particularly useful to test this concept *in vivo* (Fig. 1E-H), without the confounding effects of microbial burden.

By using a ‘sterile’ tail-fin injury model, Bernut et al. demonstrated that loss of Cftr in zebrafish induces a hyper-inflammatory response characterised by excessive neutrophil recruitment and persistence at wound sites (Bernut et al., 2020), thereby recapitulating neutrophilic inflammation characteristic of CF airway disease. Mechanistically, the phenotype involved heightened epithelial dual oxidase (DUOX)-dependent ROS signalling that amplified neutrophil chemotaxis, consistent with the role of DUOX-derived hydrogen peroxide (H_2_O_2_) gradients in leukocyte recruitment after zebrafish tissue injury (Niethammer et al., 2009). In addition, Cftr deficiency in zebrafish was also shown to impair neutrophil clearance mechanisms, including altered apoptosis and dysregulated reverse migration (Bernut et al., 2020), thereby reproducing the defective resolution of neutrophilic inflammation described in CF airways (Gray et al., 2018; Moriceau et al., 2010). Recently, the zebrafish has also proven particularly relevant to better understand the role of nuclear factor erythroid 2-related factor 2 (NRF2), a master regulator of cellular defence against oxidative stress and inflammation, in the inflammatory status associated with CF (Borcherding et al., 2019). In Cftr-deficient zebrafish, NRF2 activity appears insufficient to resolve the elevated oxidative stress, leading to exaggerated epithelial oxidative responses and increased expression of pro-inflammatory cytokines, notably Il8 and Il1b, which together drive disproportionate neutrophil recruitment (Leon-Icaza et al., 2025).

Moreover, altered Ca^2+^ homeostasis has been described in CF airway epithelial cells, and is associated with oxidative stress and exaggerated cytokine production (Rimessi et al., 2021). *In vivo*, zebrafish CF models have extended these observations by implicating voltage-gated calcium channels (VGCCs) as previously unrecognized drivers of pathological epithelial Ca^2+^ elevation, thereby linking altered Ca^2+^ influx to increased epithelial ROS production, NFκB activation and excessive neutrophil recruitment (Cornélie et al., 2025 preprint). In support of this, epithelial Ca^2+^ signals activate DUOX-dependent H_2_O_2_ production, a chemotactic cue for leukocyte recruitment after tissue damage (Razzell et al., 2013), in general zebrafish wound models.

In CF, the altered capacity of damaged tissues to heal and repair properly is an often-underestimated aspect of disease progression, despite contributing significantly to the long-term lung pathology, particularly fibrosis (Mall et al., 2024). Beyond inflammation, zebrafish offer a distinct advantage for studying repair and remodelling, owing to their regenerative capacity. Whereas wild-type larvae regenerate caudal fin tissue efficiently, Cftr-deficient zebrafish show impaired regrowth and increased tissue damage, consistent with defective inflammation resolution and repair programs (Bernut et al., 2020; Leon-Icaza et al., 2025). Although zebrafish do not recapitulate lung fibrosis per se, Leon-Icaza et al. reported signatures consistent with pathological remodelling in the caudal fin tissue, including abnormal collagen organisation, suggesting that chronic inflammatory imbalance can shift tissue programmes towards scarring-like outcomes (Leon-Icaza et al., 2025).

**Fig. 4.**
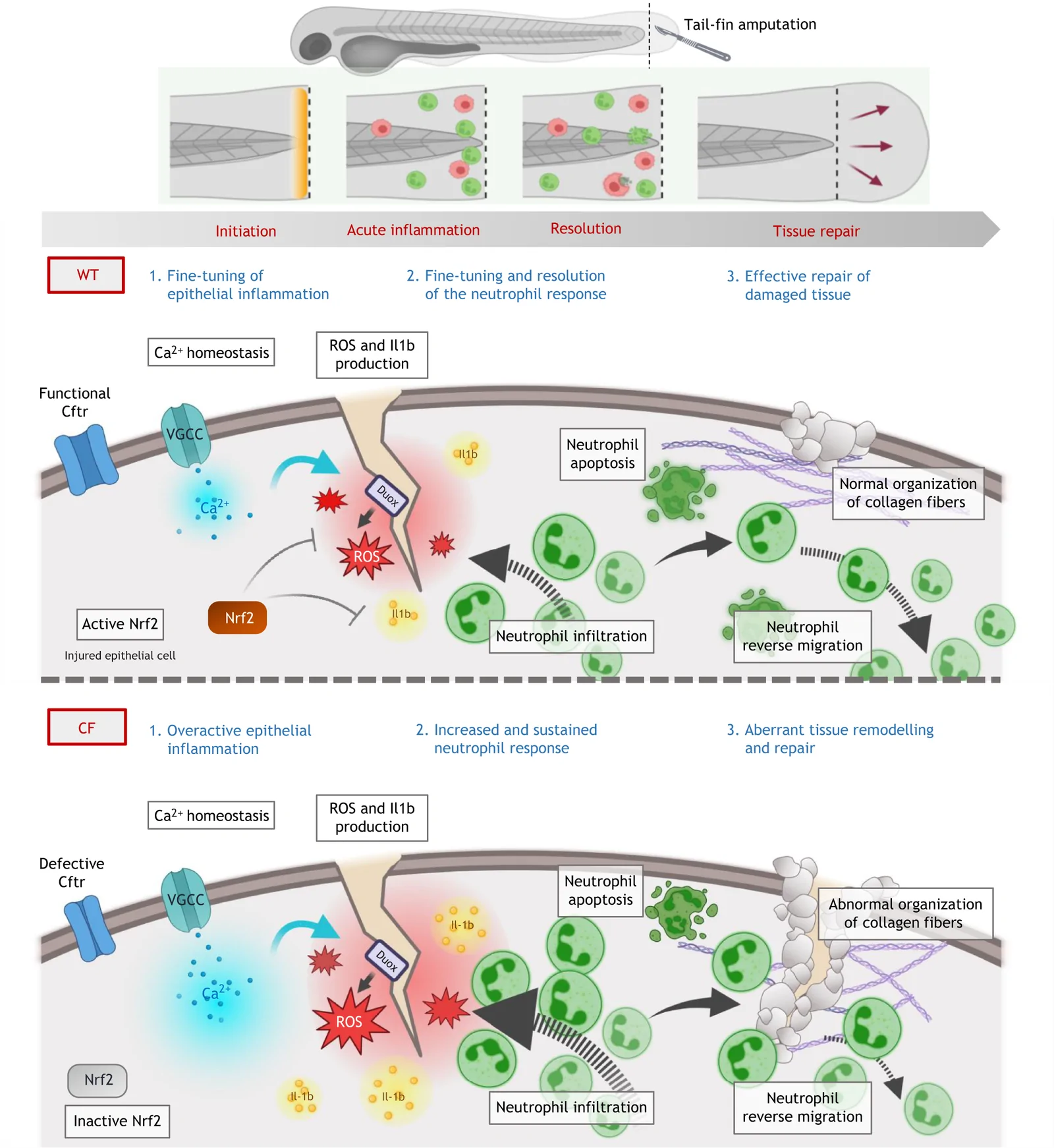
**Pathogenesis of CF-related inflammation elucidated using zebrafish CF models.** Schematic overview summarizing the spatiotemporal events triggered by Cftr depletion in wild-type zebrafish (WT), illustrating how CFTR in human orchestrates innate immune responses by regulating key signalling pathways that control inflammation and tissue repair. In Cftr-deficient zebrafish (CF), tail-fin amputation triggers an exaggerated inflammatory response characterized by dysregulated epithelial Ca^2+^ signalling, excessive production of ROS through Duox, insufficient activation of the antioxidant regulator Nrf2 and elevated Il1b release. These combined alterations drive excessive and persistent accumulation of neutrophils at the injury site. Sustained neutrophilic inflammation promotes tissue damage and, ultimately, leads to aberrant tissue repair and remodelling. Schematic illustrations were created in Biorender by Bernut, A (2026). https://BioRender.com/00zrwdj. This figure was sublicensed under CC-BY 4.0 terms.

Overall, CF zebrafish models demonstrated that CFTR dysfunction triggers excessive inflammation in the absence of infections, impairs its resolution and compromises tissue repair (Fig. 4). This highlights zebrafish as a valuable system to disentangle primary CFTR-dependent immune defects due to secondary consequences of chronic infection.

In epithelial cells, the imbalance appears reversed, with excessive ROS signalling amplifying inflammation. In zebrafish wound models, epithelial Ca^2+^ signals activate DUOX-dependent H_2_O_2_ production, a chemotactic cue for leukocyte recruitment after tissue damage (Razzell et al., 2013). In CF, altered Ca^2+^ homeostasis has been linked to oxidative stress and inflammatory signalling in airway epithelial cells (Rimessi et al., 2021), and recent zebrafish data implicate VGCCs in pathological epithelial Ca^2+^ elevation, NFκB activation and excessive neutrophilic inflammation (Cornélie et al., 2025 preprint). In parallel, insufficient NRF2 activity may fail to buffer epithelial oxidative stress, allowing ROS-dependent cytokine production and neutrophil recruitment to persist; NRF2 activation reduced epithelial ROS, dampened pro-inflammatory cytokines and improved tissue repair in Cftr-deficient zebrafish and patient-derived airway organoids (Leon-Icaza et al., 2025).

#### Dissecting CF redox imbalance with zebrafish models: from antimicrobial defence to inflammatory damage

In CF, ROS responses are dysregulated in a cell-dependent manner (Artusi et al., 2025). In phagocytes, defective ROS production compromises bacterial killing and favours infection, whereas, in epithelial tissues, excessive and unresolved oxidative signals promote inflammation and tissue damage. Cftr-deficient zebrafish models help illustrate this imbalance *in vivo*. During infection, impaired NOX2-dependent ROS production in professional phagocytes limits antimicrobial defence and favours bacterial expansion (Bernut et al., 2019). By contrast, after sterile tail-fin injury, excessive epithelial DUOX-dependent ROS signalling sustains neutrophil recruitment and delays resolution (Bernut et al., 2020). Together, these findings indicate that CFTR dysfunction disrupts the spatial control of ROS responses.

In epithelial cells, excessive ROS signalling is, in part, due to altered Ca^2+^ homeostasis (Cornélie et al., 2025 preprint) and insufficient NRF2 activity (Leon-Icaza et al., 2025), allowing ROS-dependent cytokine production and neutrophil recruitment to persist. Interestingly, NRF2 may also play a role in macrophage stress tolerance, phagocytic function and bacterial clearance; accordingly, NRF2 activation has been associated with improved macrophage bactericidal activity in infection models (Harvey et al., 2011; Zhou et al., 2022). Consistent with this, preliminary CF zebrafish data suggest that impaired NRF2 activation contributes to hypersusceptibility to *M. abscessus* infection, whereas pharmacological NRF2 activation help restore host defence (Fortuin et al., 2023).

Together, these observations support a model in which CFTR dysfunction uncouples ROS responses across cellular compartments: phagocyte ROS bursts may be insufficient for pathogen control, whereas epithelial ROS signals may persist and promote inflammatory damage. Therapeutically, this implies that redox responses should be fine-tuned rather than broadly suppressed, preserving intramacrophage antimicrobial ROS production while limiting persistent epithelial oxidative signalling.

#### Cftr-depleted zebrafish recapitulate extra-pulmonary manifestations of CF

Beyond airway disease, zebrafish models have clarified how CFTR dysfunction contributes to CF-associated pathology outside the lung. Navis and Bagnat reported that Cftr localises to the apical membrane of pancreatic ducts and that *cftr* mutant zebrafish develop progressive exocrine pancreatic destruction during larval stages, with ductal mucus accumulation, neutrophil-associated inflammation and loss of acinar tissue, thereby closely reflecting major features of CF pancreatic pathology (Navis and Bagnat, 2015). This phenotype is relevant to human CF, where ductal obstruction and inflammation drive exocrine pancreatic insufficiency and may contribute to endocrine dysfunction, including CF-related diabetes, through reduced insulin secretion, islet remodelling, local inflammation and genetic modifiers (Coderre et al., 2021; Singh and Schwarzenberg, 2017).

Zebrafish have also been used to investigate CF-associated infertility, which occurs in >95% of men with CF because of congenital bilateral absence of the vas deferens (de Souza et al., 2018). Liao et al. reported that Cftr is required for primordial germ cell migration during early development in zebrafish, with Cftr disruption impairing this process (Liao et al., 2018), providing a mechanism that may contribute to reproductive phenotypes linked to CFTR dysfunction.

Finally, Sun et al. demonstrated that Cftr-defective zebrafish exhibit primitive and definitive haematopoietic defects associated with impaired Wnt signalling (Sun et al., 2018), supporting a potential link between CFTR dysfunction and haematopoietic abnormalities. This observation may be relevant to anaemia and iron deficiency reported in subsets of people with CF, particularly in association with chronic inflammation and infection (Lobbes et al., 2022).

### Diving into drug screening: zebrafish larvae as an *in vivo* platform for CF drug discovery and assessment

Targeting chronic infection and deleterious inflammation remains a major therapeutic priority in CF, given their central role in driving lung damage and disease progression. However, the development of effective anti-infectious and anti-inflammatory therapies in CF has been hampered by the lack of suitable *in vivo* models that recapitulate the mechanistic links between CFTR dysfunction and innate immune pathogenesis, while remaining compatible with early-stage drug testing. Although mammalian CF models have provided invaluable insights, their high cost, low throughput and experimental complexity often limit their use in exploratory or medium-scale preclinical screening.

In this context, Cftr-depleted zebrafish larvae offer a unique and practical animal platform to rapidly assess therapeutic efficacy against infection and inflammation *in vivo*. Their optical transparency, genetic tractability and compatibility with pharmacological interventions at physiologically relevant concentrations enable the identification of candidate compounds and assessment of efficacy, toxicity and host-response dynamics in a whole organism. Beyond CF, zebrafish larvae have been widely used for *in vivo* small-molecule screening, including antimicrobial screens in infection models (Habjan et al., 2024) and screens for anti-inflammatory/pro-resolution properties based on neutrophil recruitment, apoptosis and reverse migration (Robertson et al., 2014). Accordingly, CF zebrafish models have been used to test diverse therapeutic strategies, particularly those targeting CF-associated infections and inflammation.

#### Therapeutic strategies for tackling infections in CF

People with CF are often exposed to prolonged and continuous antibiotic treatments to control pulmonary infections. The global rise of multidrug-resistant pathogens, particularly *P. aeruginosa* and NTM, has intensified the need for alternative or adjunctive antimicrobial strategies. Among these, phage therapy has emerged as a promising approach, particularly in CF, where pathogen persistence and resistance are major clinical challenges.

CF zebrafish models have been instrumental in evaluating the efficacy of phage-based therapies against CF-relevant pathogens. By using Cftr-deficient zebrafish, several studies demonstrated that phage treatment significantly reduces bacterial burden following infection with *P. aeruginosa* or *M. abscessus* (Cafora et al., 2019, 2021, 2022; Johansen et al., 2021). Importantly, these studies also showed that combining phages with conventional antibiotics enhances therapeutic efficacy, allowing improved infection control while potentially reducing antibiotic doses and selective pressure for resistance. In addition, by reducing bacterial burden, phage therapy can dampen infection-associated inflammation in CF zebrafish models, and data also suggest that phages modulate inflammatory responses more directly (Cafora et al., 2021, 2025).

Together, these findings highlight zebrafish as a powerful system for evaluating host−pathogen−therapy interactions in CF.

#### Strategies targeting deleterious inflammation in CF

Current anti-inflammatory treatments in CF, such as corticosteroids or high-dose ibuprofen, can reduce inflammation but are associated with significant long-term adverse effects. Consequently, there is growing interest in host-directed therapies that restore immune balance, promote inflammation resolution and limit tissue damage without compromising antimicrobial defence. CF zebrafish models have proven particularly valuable for testing such strategies *in vivo*. Multiple studies have identified compounds capable of regulating neutrophil behaviour, resolving inflammation and improving tissue repair in the context of CFTR deficiency (Cornélie et al., 2025 preprint; Bernut et al., 2020; Le Moigne et al., 2022; Leon-Icaza et al., 2025).

One notable example is tanshinone IIA, which was initially identified in an *in vivo* zebrafish screen for pro-resolution molecules, where live imaging showed that it accelerates neutrophil clearance from inflammatory sites by enhancing their reverse migration and apoptosis (Robertson et al., 2014), but its precise molecular target in this context has not been definitively established. Then, the compound was similarly shown to effectively resolve sustained neutrophilic inflammation in Cftr-deficient zebrafish, by promoting both neutrophil apoptosis and reverse migration (Bernut et al., 2020). The anti-inflammatory effect of tanshinone IIA is also accompanied by improved tissue repair in CF zebrafish, positioning this compound as a promising candidate for mitigating CF-related inflammatory damage (Bernut et al., 2020).

As discussed above, zebrafish studies have reinforced the concept that NRF2 signalling is insufficiently activated in CF, contributing to excessive oxidative stress and inflammatory injury (Leon-Icaza et al., 2025). By using Cftr-depleted zebrafish and CF patient-derived airway organoids, NRF2 activators, most notably curcumin, were shown to reduce epithelial oxidative stress, dampen proinflammatory cytokine production, attenuate neutrophil-driven inflammation and improve tissue repair (Leon-Icaza et al., 2025). Preliminary CF zebrafish data suggest that curcumin-mediated NRF2 activation also improves the outcome of *M. abscessus* infection by supporting host defences while reducing inflammatory damage (Fortuin et al., 2023). These findings identify NRF2 as an interesting therapeutic target and support curcumin as a candidate for host-directed anti-inflammatory and anti-infectious therapy in CF.

In addition to redox signalling, epithelial Ca^2+^ homeostasis has emerged as a critical regulator of inflammatory responses in CF. Recent CF zebrafish data indicate that epithelial VGCCs drive pathological Ca^2+^ elevation, oxidative stress, NFκB activation and excessive neutrophilic inflammation, and that direct inhibition VGCCs with the FDA-approved cardiovascular disease drug verapamil reduces epithelial oxidative stress and neutrophil recruitment (Cornélie et al., 2025 preprint). Verapamil is also of interest in infectious contexts because, beyond its action on Ca^2+^ channels, it has been reported to potentiate the activity of antimycobacterial antibiotics by inhibiting bacterial efflux mechanisms, including in *M. abscessus* studies (Gupta et al., 2015). These findings support drug-repurposing strategies targeting epithelial Ca^2+^ to alleviate pathological inflammation.

Targeting NRF2 signalling and/or epithelial Ca^2+^ homeostasis shows promising potential as both strategies limit CF-associated inflammation. NRF2 activation may also support antimicrobial host defence, whereas targeting pathological epithelial Ca^2+^ signalling appears primarily relevant for limiting excessive inflammation. Similarly, pro-resolution compounds, such as tanshinone IIA, are attractive because they appear to promote neutrophil apoptosis and reverse migration rather than simply blocking neutrophil mobilisation. This distinction is important, as enhancing the clearance of neutrophils after their antimicrobial function may be less detrimental during infection than preventing their recruitment altogether; however, this hypothesis remains to be tested directly in infected models.

#### The importance of *in vivo* validation: lessons from roscovitine

Importantly, zebrafish models have also revealed unexpected detrimental effects of candidate therapies that would have been difficult to predict using *in vitro* systems alone. For example, although roscovitine exhibits anti-inflammatory and pro-resolution properties and enhances intracellular bacterial killing in human CF macrophages, its administration worsened *M. abscessus* infection in both zebrafish and mouse models (Le Moigne et al., 2022). Mechanistically, roscovitine interferes with DUOX- and/or NADPH oxidase-dependent ROS production, reducing epithelial oxidative signalling and neutrophil recruitment. Although this inference reduces inflammation, it simultaneously compromises formation and maintenance of granulomas, structures essential for containment of mycobacterial infections, leading to extracellular bacterial expansion and disease exacerbation. This example illustrates that anti-inflammatory activity cannot be evaluated independently from host-defence mechanisms. Roscovitine, therefore, highlights why candidate therapies should be evaluated in whole-organism models to capture the full spectrum of drug effects on immunity, infection and tissue integrity in CF.

In CF, the therapeutic challenge is not simply to suppress inflammation but to preserve antimicrobial functions while preventing their progression into chronic tissue-damaging responses. In this context, strategies that rebalance redox and inflammatory signalling without broadly blocking immune recruitment may be particularly valuable, i.e. approaches targeting NRF2 signalling and epithelial Ca^2+^ homeostasis.

Collectively, zebrafish CF models provide a powerful preclinical platform to accelerate drug discovery, identify therapeutic targets and uncover consequences of candidate treatments. By integrating *in vivo* infection biology, innate immune dynamics and tissue repair outcomes, zebrafish uniquely complement mammalian models and human-derived cellular systems, helping to bridge the gap between mechanistic insight and translational application in CF.

## Limitations and perspectives of the zebrafish model in CF research

Despite its many advantages, the zebrafish model has inherent limitations that must be carefully considered when interpreting findings and assessing translational relevance to CF. Foremost among these is the absence of lungs, which represents a major constraint given that CF is primarily a pulmonary disease. Consequently, hallmark features of CF lung pathology, such as spontaneous airway infections, bronchiectasis, mucus plugging and progressive decline in lung function, cannot be directly modelled in zebrafish. Although zebrafish enable detailed investigation of CFTR-dependent epithelial and immune dysfunction, they do not fully recapitulate the complex microenvironment of the human CF airway, including chronic polymicrobial colonisation and long-term remodelling of the respiratory epithelium. Additional anatomical and physiological differences further limit the modelling of specific CF manifestations. For example, the zebrafish reproductive system differs substantially from that of humans, restricting its ability to fully model CF-related male infertility caused by congenital bilateral absence of the vas deferens. Likewise, while the remarkable regenerative capacity of zebrafish is a powerful asset for studying tissue repair, it may represent a limitation for modelling fibrotic diseases such as CF, where irreversible scarring and progressive tissue destruction are central features. Extensive regenerative mechanisms in zebrafish may, therefore, mask or counteract fibrotic processes that are prominent in human CF pathology.

Accurate modelling of immune responses is also critical for understanding CF pathogenesis. As a vertebrate, zebrafish possess a highly conserved immune system, particularly with respect to innate immunity. However, differences remain. Notably, while zebrafish larvae display a fully functional innate immune system, adaptive immunity matures later, typically ∼4-6 weeks post fertilisation (Renshaw and Trede, 2012). This makes larval zebrafish particularly well suited to study early innate immune responses, neutrophil and macrophage dynamics, host−pathogen interactions and acute inflammation, but less appropriate for modelling CF processes that involve adaptive immune components, such as chronic antigen exposure, lymphocyte-dependent immune regulation or long-term host−pathogen adaptation (Bruscia and Bonfield, 2022). Furthermore, many CF-associated pathological changes, including chronic colonisation, persistent inflammation, progressive tissue remodelling, fibrosis and organ dysfunction, reflect long-term interactions between infection, inflammation and tissue repair. Because most larval zebrafish studies are performed over hours to days, they provide exceptional access to early cellular mechanisms but remain limited for modelling chronic disease evolution. Some of these limitations can be addressed by using juvenile or adult zebrafish, in which adaptive immunity is mature and long-term host responses can be investigated. However, adult studies are associated with increased ethical and husbandry constraints, reduced throughput and loss of the natural optical transparency that makes larvae so powerful for live imaging. Transparent adult lines, such as the *Casper* zebrafish (White et al., 2008), can partially overcome this imaging limitation and may facilitate longitudinal studies of immune and tissue dynamics in older animals.

Finally, although CFTR is highly conserved between zebrafish and humans, overall genetic homology is lower than in mammalian models, such as mice. To date, most zebrafish CF studies have relied on Cftr-depleted models (stable knockouts or morphants), which capture loss-of-function phenotypes but do not reflect the diversity of CF-causing mutations or their mutation-specific effects on protein processing, channel activity or therapeutic responsiveness. The development of zebrafish lines carrying patient-relevant CFTR mutations would, therefore, represent an important advance. Such models would enable investigation of genotype-specific mechanisms and evaluation of variant-specific therapies, while complementing existing mammalian and human-derived cellular systems.

## Concluding remarks

Over the past decade, zebrafish have emerged as a highly relevant and complementary model in CF research, bridging the gap between *in vitro* systems and mammalian models. While the absence of lungs remains an important limitation, increasing evidence indicates that CFTR-dependent epithelial and innate immune functions are conserved across tissues in zebrafish and mammals. As a result, key CF-associated defects, such as impaired antimicrobial immunity, dysregulated inflammation and abnormal tissue repair, can be modelled and mechanistically dissected in this system. Beyond airway-centred pathology, zebrafish also offer opportunities to investigate extra-pulmonary manifestations of CF, which remain comparatively understudied despite their impact on patients' quality of life and their incomplete resolution by current therapies, including CFTR modulators. Looking forward, the greatest value of zebrafish will probably lie not in replacing existing CF models but in strengthening the coordinated use of complementary models. Patient-derived organoids and primary cells provide human genetic and epithelial relevance, but mammalian models remain essential for modelling lung architecture and chronic disease. Zebrafish, however, uniquely combine genetic and functional conservation of CFTR, a highly tractable innate immune system, optical transparency for intravital imaging and compatibility with *in vivo* drug screening. These features enable real-time analysis of host−pathogen interactions, inflammatory dynamics, tissue repair and organ-specific consequences of CFTR dysfunction in an intact vertebrate at scale.

Future work should, therefore, focus on strengthening the translational interface between zebrafish, human-derived systems and mammalian models. The generation of zebrafish lines carrying patient-relevant CFTR variants, combined with high-resolution imaging, single-cell approaches and phenotypic screening, could help define how specific mutations alter infection susceptibility, inflammatory resolution, tissue remodelling and therapeutic responsiveness *in vivo*. Such cross-model validation will be particularly important for host-directed therapies aimed at fine-tuning immune responses, where efficacy against inflammation must be balanced against preservation of antimicrobial defence, as well as for therapeutic strategies targeting extra-pulmonary manifestations that remain insufficiently addressed.

In this broader modelling landscape, zebrafish provide a distinctive opportunity to uncover primary CFTR-dependent mechanisms, identify therapeutic targets and guide the selection of candidate compounds for further validation in more complex systems. By combining mechanistic accessibility with whole-organism physiology, CF zebrafish models can help accelerate our understanding of CF pathogenesis and support the development of innovative therapies for people with CF.
